# Characterization of Functional and Structural Integrity in Experimental Focal Epilepsy: Reduced Network Efficiency Coincides with White Matter Changes

**DOI:** 10.1371/journal.pone.0039078

**Published:** 2012-07-12

**Authors:** Willem M. Otte, Rick M. Dijkhuizen, Maurits P. A. van Meer, Wilhelmina S. van der Hel, Suzanne A. M. W. Verlinde, Onno van Nieuwenhuizen, Max A. Viergever, Cornelis J. Stam, Kees P.J. Braun

**Affiliations:** 1 Rudolf Magnus Institute of Neuroscience, University Medical Center Utrecht, Utrecht, The Netherlands; 2 Image Sciences Institute, University Medical Center Utrecht, Utrecht, The Netherlands; 3 Department of Clinical Neurophysiology, VU University Medical Center, Amsterdam, The Netherlands; Kaohsiung Chang Gung Memorial Hospital, Taiwan

## Abstract

**Background:**

Although focal epilepsies are increasingly recognized to affect multiple and remote neural systems, the underlying spatiotemporal pattern and the relationships between recurrent spontaneous seizures, global functional connectivity, and structural integrity remain largely unknown.

**Methodology/Principal Findings:**

Here we utilized serial resting-state functional MRI, graph-theoretical analysis of complex brain networks and diffusion tensor imaging to characterize the evolution of global network topology, functional connectivity and structural changes in the interictal brain in relation to focal epilepsy in a rat model. Epileptic networks exhibited a more regular functional topology than controls, indicated by a significant increase in shortest path length and clustering coefficient. Interhemispheric functional connectivity in epileptic brains decreased, while intrahemispheric functional connectivity increased. Widespread reductions of fractional anisotropy were found in white matter regions not restricted to the vicinity of the epileptic focus, including the corpus callosum.

**Conclusions/Significance:**

Our longitudinal study on the pathogenesis of network dynamics in epileptic brains reveals that, despite the locality of the epileptogenic area, epileptic brains differ in their global network topology, connectivity and structural integrity from healthy brains.

## Introduction

Widespread, bilateral structural and functional abnormalities have been reported in people with epilepsy, even when the epileptic syndrome is localization-related, idiopathic or cryptogenic, and the brain appears normal on conventional magnetic resonance imaging (MRI) [1], [2]. Such tissue damage distant from the epileptogenic zone has been observed in both white [2], [3] and gray matter [4], [5], [6].

These subtle progressive changes in tissue integrity that are mostly undetectable with conventional MRI and extend outside the margins of the primary epileptogenic area [7], are presumed to be the result of recurrent seizure propagation [8]. This is thought to play a crucial role in epilepsy as these changes may significantly modify the global structural and functional network topology [9].

The characterization of brain networks has been greatly facilitated by the exact mathematical formalism provided by graph theoretic analysis [10]. In graph analysis a network is represented as a set of vertices and edges. Different network classes can be characterized based on the type of configuration vertices and edges. More specifically, three network classes, namely regular, small-world, and random, can be differentiated based on their clustering, a property of segregation, and shortest path, a property of integration. High values of both clustering and shortest path are found in regular networks. At the other extreme, if the nodes are randomly interconnected, both measures are low. Low values of shortest path and high values of clustering reflect a small-world network topology, which is proposed to be an optimal network configuration for global information transfer and local processing [11].

Significant changes in clustering and shortest path have been reported, based on electrophysiology, in people with epilepsy [12]. Several studies have shown that during seizures the global network shifts from a small-world topology towards a more regular topology. Based on these findings, it has been hypothesized that interictal epileptic functional networks have a more random, that is, opposite from regular, topology [13]. More recently, interictal brain networks in individuals with focal, most often temporal, epilepsy were characterized functionally and structurally and compared to control networks [14], [15], [16], [17], [18], [19]. Results, however, are not unequivocal. Both an increase in clustering and shortest path [14], [17], [19], and a decrease in these network properties have been described in the interictal epileptic state [15]. In addition, a decreased clustering and increased path length has been reported [16]. Methodological differences or incomparability of study populations could attribute to the different network topologies found. Incomparability of natural history is a well-known threat for most observational studies [20]. Specific confounders in the study of network differences in patients with temporal epilepsy include a possible history of febrile seizures, age at onset, duration of epilepsy at time of inclusion, the presence of dual pathology [21], and the use of antiepileptic drug treatment. In particular the latter may contribute to changes in network topology as antiepileptic drugs can affect brain development with long-term neurological consequences [22]. Adequate separation of these extraneous influences from the effect that spontaneous recurrent seizures have on the network topology is very difficult in a clinical setting.

A preclinical study in a well-characterized animal model of neocortical epilepsy allows to assess – both spatially and temporally – the effect of focal epilepsy on the interictal network topology, without the aforementioned confounders, in two groups that are identical except for the occurrence of spontaneous recurrent seizures. In this study we aimed to characterize the effect of spontaneous recurrent seizures occurring from a focal epileptogenic area on the functional network configuration. To that aim, we measured the spatiotemporal evolution of changes in interictal brain networks in a rat model of neocortical focal epilepsy by means of serial *in vivo* resting-state functional MRI (rs-fMRI) acquisition over ten weeks of time. Measurement of spontaneous low-frequency blood oxygenation level-dependent (BOLD) fluctuations with rs-fMRI allows the assessment of changes in signal synchronization at the level of hemispheres, regions or voxels. Functional networks are based on neuronal signal synchronizations underlying brain communication. Our longitudinal setup of rs-fMRI acquisition provides information on the stability of the network topology in brains subjected to focal epilepsy. We had the following hypotheses: (a) functional interictal networks shift towards a more random topology; (b) these shifts are consistent over time; (c) network changes are associated with changes in intra- as well as interhemispheric functional connectivity.

An additional unsolved issue is the relation between changes in functional connectivity, network topology, and the microstructural white matter damage, which is known to occur in focal epilepsy [2], [3]. We therefore included diffusion tensor imaging (DTI), which enables the assessment of white matter structural reorganization non-invasively. This interrogation of white matter structure *in vivo* is based on measurement of the diffusion process that is effectively captured as a diffusion tensor. The most frequently used diffusion tensor parameter is the fractional anisotropy, a measure of preferred directionality of diffusion within a voxel. DTI has been indicated to be much more sensitive in detecting microstructural alterations in white matter as compared to conventional structural MRI [3], [23], [24]. We hypothesized that epilepsy-induced network topology and functional connectivity alterations are accompanied by remote changes in tissue water diffusion properties in bilateral white matter.

Unravelment of the extent and time course of shifts in network topology and the relation with structural white matter integrity in a controlled setting may provide new insights into the pathogenesis of focal epilepsy and its consequences for brain function.

## Results

### Epilepsy model

**Figure 1:**
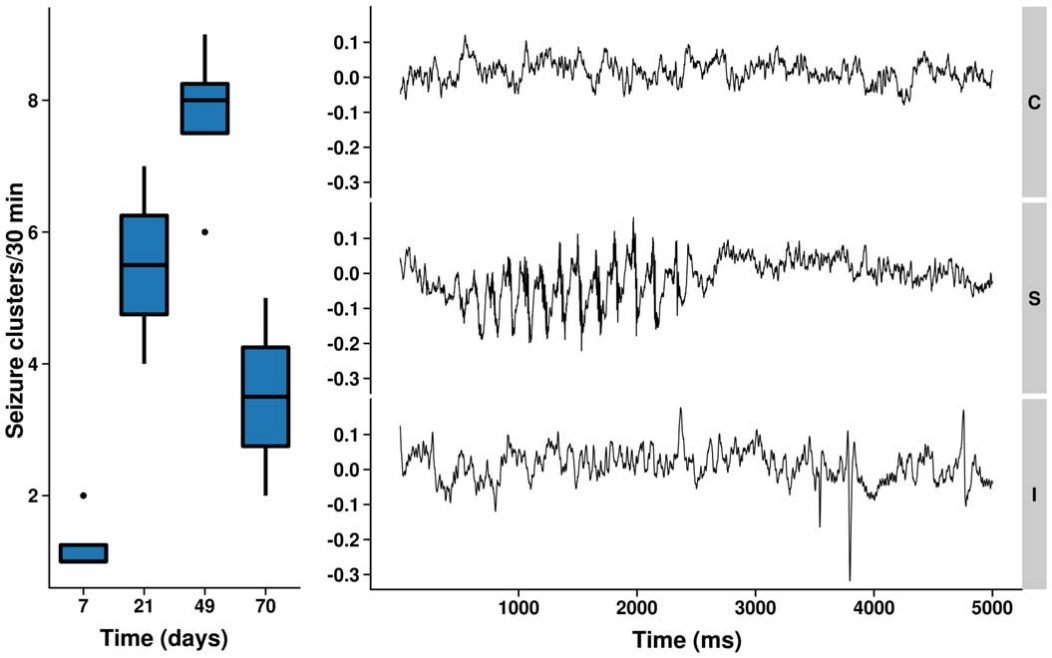
Spontaneous seizures during study follow-up. Left: Number of seizure clusters scored during 30 minutes observation epochs. Right: Representative 5 seconds EEG selected from ten minutes recordings from a control rat at 1.0% isoflurane (C); and from an epileptic rat seven weeks post induction, interictally at 1.0% isoflurane (I), and during a motor seizure at 0% isoflurane (S). The interictal EEG is characterized by a similar baseline rhythm as the control EEG with infrequent interictal spikes. The ictal EEG involved high amplitude rhythmic spike series (only one shown).

We used a well known focal epilepsy tetanus toxin rat model [25], [26], with the right primary motor cortex as injection side [27], [28]. The tetanus toxin injection induced frequent, mild, but persistent facial motor seizures in all animals. Spontaneous, well-tolerated seizures occurred in clusters, and persisted for multiple weeks. Seizures started around one week after tetanus toxin injection, with a peak in frequency (on average 8 seizure clusters per 30 minutes) at seven weeks, followed by a decline towards the latest time point (Figure 1, left). Seizure clusters typically lasted from ten seconds to three minutes. In addition, seizures specifically occurred during onset of and recovery of anesthesia, which was confirmed by electroencephalography (EEG) recordings, showing high amplitude rhythmic spiking on EEG at 0% isoflurane anesthesia, not related to motion artifacts (Figure 1, right). At 1% isoflurane concentrations, clinical seizures were absent and no ictal discharges were recorded with EEG, although interictal spikes were occasionally observed in epileptic rats. Five animals that developed repeated generalized tonic-clonic seizures or status epilepticus in the second week after induction were excluded from further analysis. Four rats developed aggressive behavior after the second week and were housed individually. Normal body weight gain over time was slightly reduced in the tetanus toxin treated rats, with weight values being lower only at day 21 (control group: 415±20 g; epilepsy group: 381±31 g (mean ± SD); *p* = 0.03).

Structural damage at the injection site was inspected on the anatomical images. T_2_-weighted scans are sensitive for edematous brain alterations as a result of frequent seizure propagation [29]. We found hypointensities at day 7, but not at the subsequent time points.

### Resting-state functional MRI

#### Graph analysis of functional networks

**Figure 2:**
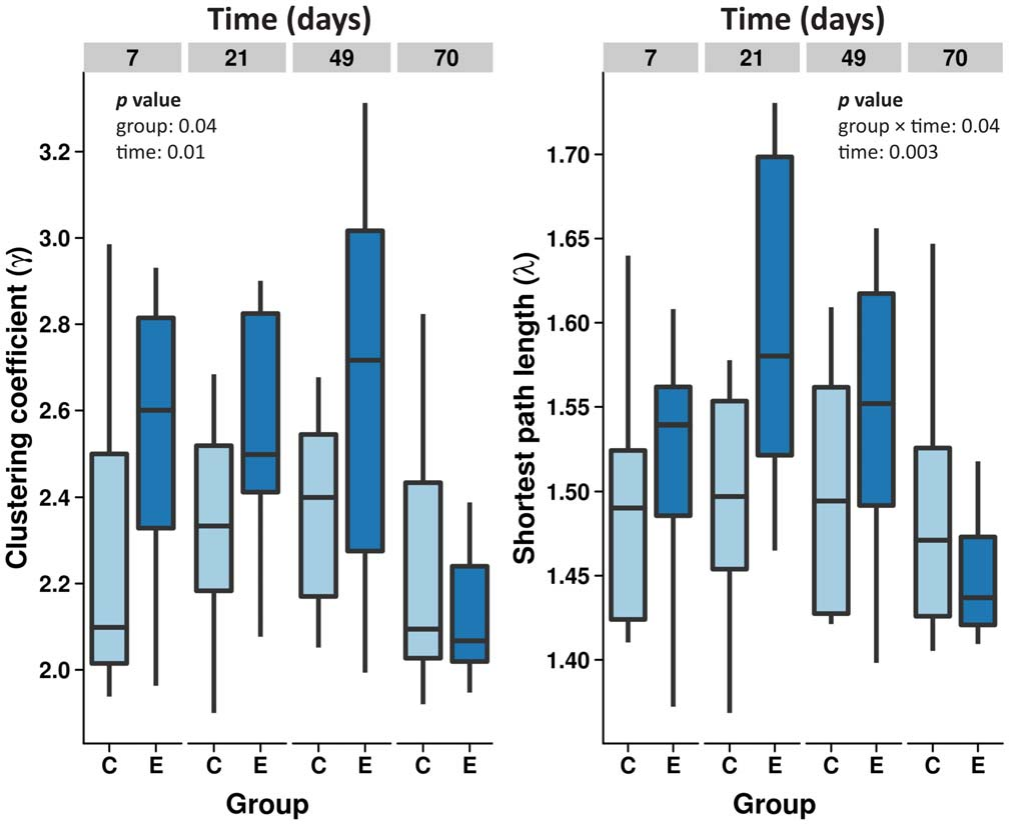
Graph analysis results. The temporal pattern of global brain functional network characteristics, i.e. the normalized clustering coefficient (γ) and normalized characteristic shortest path length (λ), at days seven, 21, 49 and seventy in control (C) and epilepsy animals (E). No significant changes over time were found for the control group. The epilepsy networks were characterized by increased γ and λ up to seven weeks. Differences between interictal networks and controls were absent at the last time point. Statistical significance obtained from the linear mixed model analysis is indicated for factors with p<0.05.

The normalized clustering coefficients (γ) and normalized characteristic shortest path lengths (λ) in the brain network at seven, 21, 49 and seventy days are depicted in Figure 2 for both experimental groups.

Based on the repeated linear mixed model fits, we found a significant γ difference for group (*p* = 0.04) and time (*p* = 0.01). γ increased over time in the epilepsy group (*p* = 0.009), but not in the control group. We found a significant λ interaction between group and time (*p* = 0.02). In the epilepsy group, but not the control group, λ increased over time (*p* = 0.003). In the epilepsy group, differences in γ and λ normalized over time, reaching control values at ten weeks after epilepsy induction.

#### Region-of-interest analysis of functional connectivity

**Figure 3:**
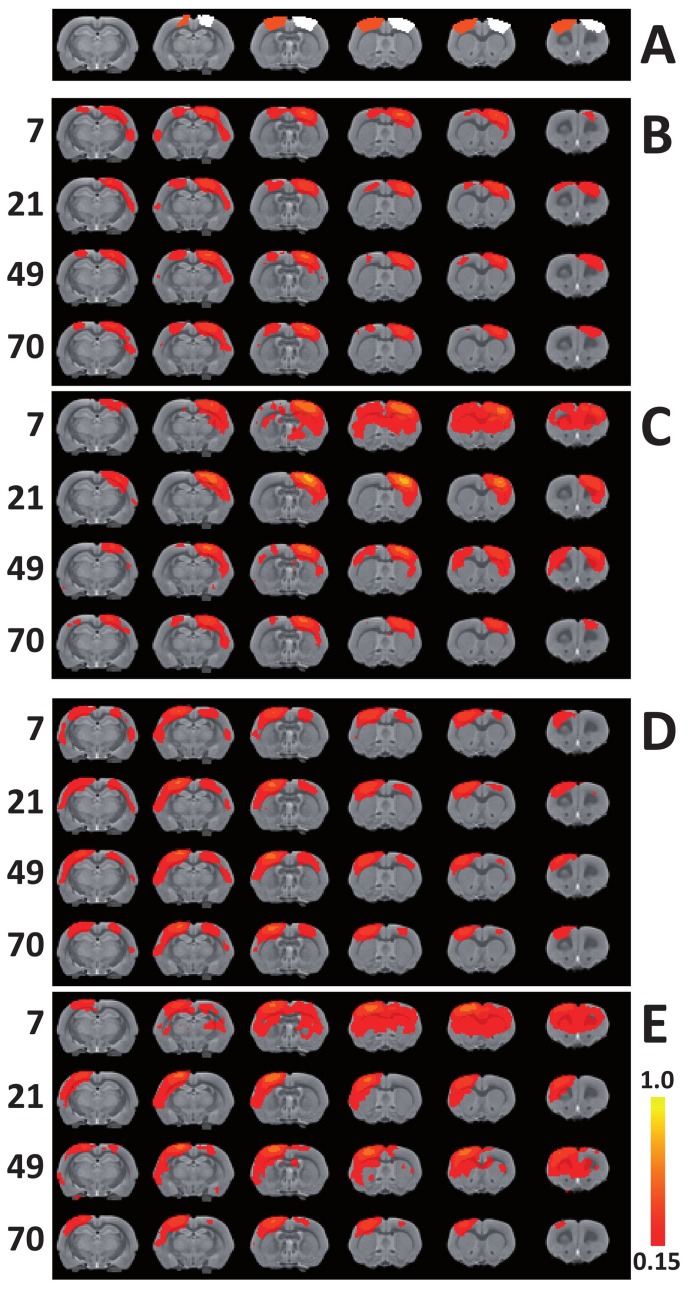
Spatial functional connectivity maps for the bilateral sensorimotor cortices. Functional connectivity maps of right and left sensorimotor cortices with the rest of the brain. Right (ipsilateral) (white) and left (contralateral) sensorimotor cortical ROIs (orange) overlaid on coronal slices from a T_2_-weighted rat brain template (**A**). Maps of functional connectivity with right, ipsilateral (**B**: control group; **C**: epilepsy group) and left, contralateral sensorimotor cortex (**D**: control group; **E**: epilepsy group). Time points (days) are shown on the left. Functional connectivity (*z*') values range from 0.15 to 1.0. The control group shows strong and consistent functional connectivity between both sensorimotor cortices at all time points. In the epileptic brain, functional connectivity was elevated at day seven, extending into the adjacent secondary somatosensory and medial cingulate cortices, the subcortical caudate putamen, and contralateral homologous areas. At day 21 after epilepsy induction, interhemispheric functional connectivity from both ROIs was clearly reduced, which recovered at the later time points.

Mean functional connectivity maps of the left and right sensorimotor cortices (regions-of-interest (ROIs) outlined in Figure 3A) with the rest of the brain clearly demonstrate strong intrahemispheric functional connectivity enhancement within the contiguous cortex and subcortical caudate putamen in the injected hemisphere (Figure 3C), and to a lesser extent in the contralateral hemisphere (Figure 3E), in the epilepsy group up to seven weeks. Interhemispheric functional connectivity of both sensorimotor ROIs and the contralateral hemisphere was reduced at 21 days after epilepsy induction, and recovered thereafter (Figure 3).

**Figure 4:**
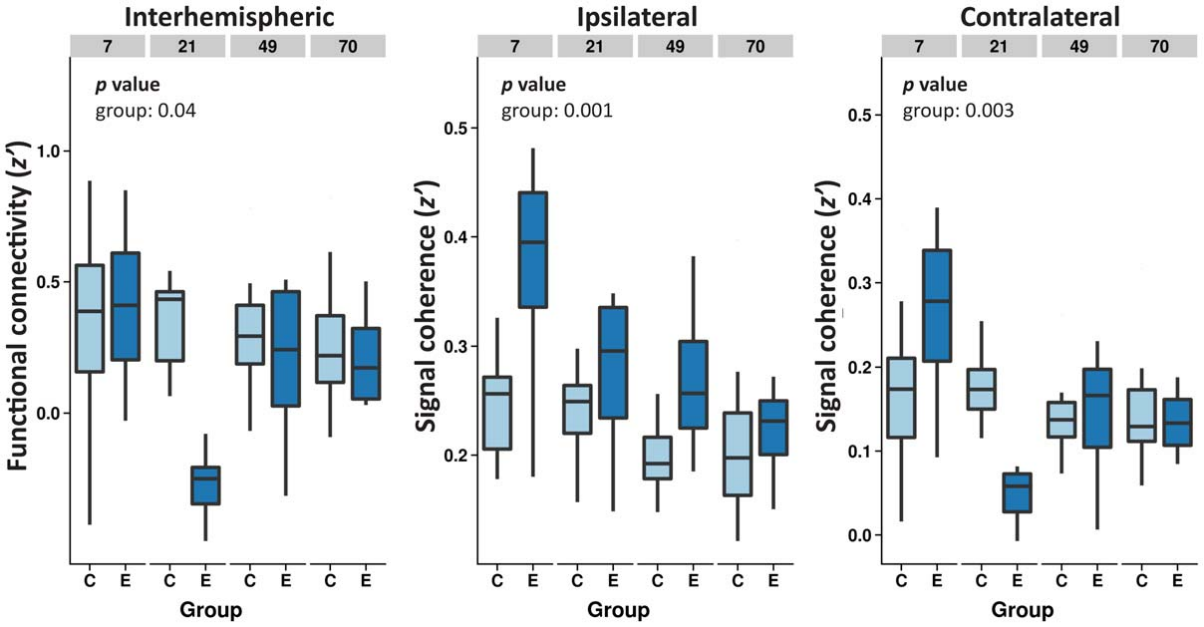
Functional connectivity between and within hemispheres. Interhemispheric functional connectivity (as normalized correlation: *z*') between the ipsilateral and contralateral sensorimotor cortical regions (left graph), and intrahemispheric functional connectivity (as volume of voxels connected with the sensorimotor ROI within the same hemisphere) for the ipsilateral (middle graph) and contralateral hemisphere (right graph) in the controls (C) and epilepsy (E) groups. Interhemispheric functional connectivity was stable over time in controls, but lowered at day 21 in the epilepsy group. The intrahemispheric functional connectivity was increased in the epilepsy group up to seven weeks, but declined to control levels at the latest time point. Statistical significance obtained from the linear mixed model analysis is indicated for factor with p<0.05.

The inter- and intrahemispheric functional connectivities of both the left and right cortical sensorimotor areas remained stable over time in the control animals (Figure 4). In contrast, the overall interhemispheric functional connectivity was diminished in the epileptic group (group effect: *p* = 0.04; Figure 4). This was due to the reduction at day 21 (*p* = 0.01), where seven out of eight epileptic rats had strong negative *z*' values (anti-correlations). Increased intrahemispheric functional connectivity was found in both hemispheres in epilepsy rats (group effects: *p* = 0.001 (ipsilateral), *p* = 0.003 (contralateral)), which returned to control levels at day 70.

### Diffusion tensor imaging

**Figure 5:**
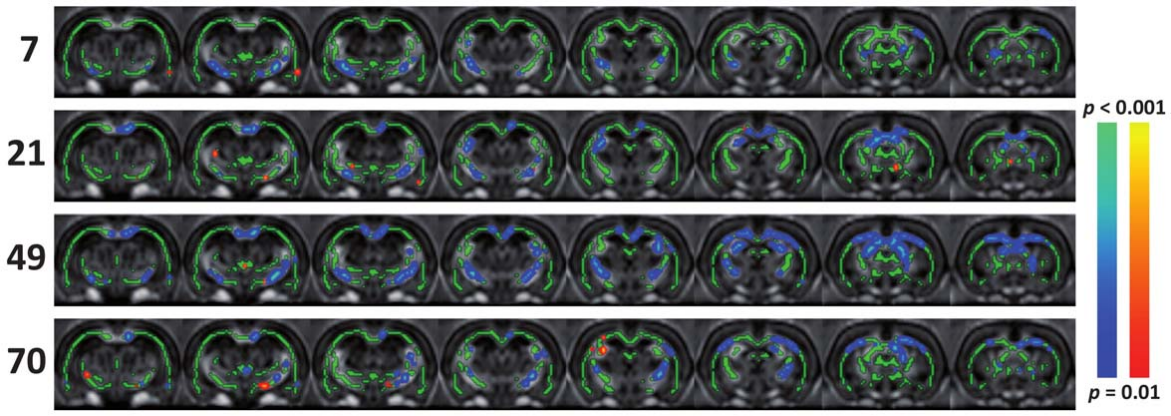
Tract-based spatial statistics results. Tract-Based Spatial Statistics output, illustrating significant differences in white matter fractional anisotropy (FA) values in epileptic brain as compared to controls (blue: reduced; red: increased. Color codes represent *p*<0.01 – *p*<0.001; false discovery rate corrected), overlaid on average FA maps of adjacent coronal rat brain slices. White matter skeleton is shown in green. Time points (days) are shown on the left.

Focal epilepsy resulted in significant lower fractional anisotropy (FA) values in white matter, which was observed as early as one week post induction. Figure 5 shows results from the Tract-Based Spatial Statistics (TBSS) analysis for all time points. Significant FA reduction was found in the ipsilateral internal and external capsules. At later time points, reduced FA was found in all major white matter bundles in both hemispheres, most profoundly at the seven week time point which recovered thereafter. We found no abnormal signal enhancement on T_2_-weighted images (data not shown).

**Figure 6:**
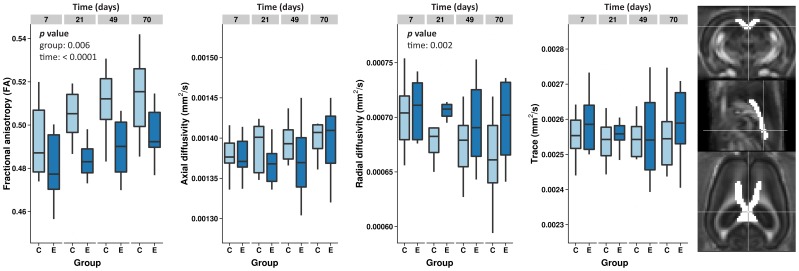
Corpus callosum diffusion tensor imaging measures. Fractional anisotropy (FA), axial and radial diffusivity, and ADC_trace_ values in the medial corpus callosum at each time point for the control and epilepsy groups. FA increased gradually in control animals. In epilepsy rats, this age-related increase was delayed and absolute values were lower. None of the other three parameters showed significant group or group × time interaction differences. The corpus callosum ROI is overlaid on the FA template (ROI in white; shown on slices in three orthogonal directions) (most right). Statistical significance obtained from the linear mixed model analysis is indicated for factor with p<0.05.

In addition to the whole brain voxel-wise white matter statistics, ROI analysis was performed in the medial corpus callosum (delineated in Figure 6, most right). The average FA, trace of the apparent diffusion coefficient (ADC_trace_), and axial and radial diffusivity values at each time point for both groups are shown in Figure 6. Callosal FA values in the epilepsy group were lower as compared to controls at all time points (*p* = 0.006). Increases in FA were seen over time for both groups (*p*<0.0001). ADC_trace_, axial and radial diffusivity values did not differ between groups, but a significant time effect was found for radial diffusivity in both groups (*p* = 0.002). ADC_trace_ and axial diffusivity did not change over time.

## Discussion

In this study, we applied serial rs-fMRI and DTI in a rat model of refractory focal neocortical epilepsy to longitudinally characterize functional connectivity, global network configuration and white matter integrity associated with chronic epilepsy.

By acquiring whole brain connectivity data at multiple time points after epilepsy induction, we gained new insights in the temporal profile of interictal network topology. Our main findings are that (a) graph-based network properties γ and λ increase in the interictal state, indicating a more regular brain network configuration; (b) interhemispheric functional connectivity in epileptic brain decreases, whereas intrahemispheric functional connectivity increases in both hemispheres; and (c) concomitantly, structural white matter integrity is disrupted, not restricted to bundles in close vicinity to the epileptogenic focus, but including the main commissural structure, the corpus callosum.

The importance of network organization for seizure spread in epilepsy has been emphasized in multiple modeling studies [30], [31], [32], [33] and confirmed with EEG [17], [34], magnetoencephalography (MEG) [14], rs-fMRI [15], [18] and DTI [16]. In this study we hypothesized that the focal epileptic brain, during seizure-free periods, would have a state of increased susceptibility to seizure generation and spread, which has been proposed to be associated with a more random network organization [30], [35]. This hypothesis is largely based on the previously reported shift towards a more ordered network configuration during seizures, as compared to the interictal states in temporal lobe and absence epilepsy syndromes [12], [36]. Interictal network topology in cortical focal epilepsy, however, has until now not been directly compared to the healthy control network state. Our study in a neocortical focal epilepsy model demonstrates that the interictal epileptic brain is characterized by a more ordered configuration, with higher γ and λ as compared to the healthy brain. In contrast with previous global network epilepsy studies, we assessed the network topology serially. This provided unique insights in the unknown interictal neuronal network dynamics. Most importantly, the affected network topology recovers within a time span of ten weeks. This recovery coincides with the reduced seizure frequency. This substantial alterations in network topology could be one of the explanations of the conflicting results found in previous cross-sectional studies. We speculate that the increased intrahemispheric functional connectivity is related to local neuronal sprouting instead to distant functional interactions in the interictal brain status. However, future research is required to address this issue using longitudinal (immuno) histopathological experiments with stainings for both myelination and axonal integrity, preferably at multiple time points after focal epilepsy induction.

The longitudinal changes in global network properties closely matched with the patterns of the intrahemispheric functional connectivity. We suppose a relationship between the increase in both γ and intrahemispheric functional connectivity as γ is a measure of the degree to which functional nodes tend to cluster together. The increased γ corroborates with previous seizure-free network findings in patients with absence epilepsy [14] and temporal neocortical epilepsy [17], where higher interictal γ was most pronounced in the EEG and MEG delta bands. This increase is in line with the idea that neural disturbances are correlated with changes in functional network organization [37], [38] and probably occur in a wide range of epilepsy syndromes. On the other hand, a different temporal lobe epilepsy (TLE) study reported interictal functional networks with lower λ [15]. This dissimilarity with our findings may be explained by differences between location in focus (temporal versus primary motor cortex), duration of epilepsy (more than 13 years versus weeks), use of antiepileptic drugs, and differences between network organization in humans and rats.

λ is a measure of the ability to rapidly combine specialized information from distributed brain areas [10]. The observed increase in interictal λ in our study was accompanied by a decrease in interhemispheric functional connectivity, which points toward a relationship between these parameters. The largest deviation in both measures was found at day 21, which subsequently normalized towards the latest time point. Although functional network paths represent sequences of statistical associations, making an analogy with the structural network difficult, modeling has shown that functional resting-state networks largely overlap with the underlying structural network [39]. The disturbed corpus callosum integrity may be held responsible for the decrease of both λ and interhemispheric connectivity. The potential relationship between increased λ and reduced interhemispheric functional connectivity is also in agreement with a recent study, that reported a striking loss of interhemispheric low-frequency blood oxygenation level-dependent (BOLD) signal correlations after corpus callosotomy, while intrahemispheric networks were preserved [40]. However, whether the integrity of the connecting white matter between the two hemispheres is truly related to the decreased interhemispheric functional connectivity needs further study, for example using computational models [41].

Knowledge of the status of the epileptic brain's structural connections is important as the above described global network alterations could be caused by white matter abnormalities, such as disruption of association fibers that may underlie the presumed long-distance functional connections. Our structural analyses add to the previous DTI epilepsy work by comparing controls to drug-naïve subjects, longitudinally. The temporal pattern of white matter FA changes resembled the temporal change in λ, suggesting a close relationship: widespread abnormalities at day 21 and 49, and recovery at ten weeks. The TBSS results indicated that the corpus callosum was substantially affected, which was confirmed by specific ROI analysis.

The diffuse structural abnormalities found in both hemispheres with DTI are in agreement with previous partial epilepsy DTI studies (for overview see: [2], [3]). Possible explanations for both the structural damage and associated changes in functional network organization include synaptic changes, neuronal death or glial cell damage. Synaptic alterations have been observed during the process of secondary epileptogenesis [42], suggesting that the anatomically distant areas undergo a physiological change consequent to neuronal alterations at the primary epileptogenic zone [43].

In addition, experimental studies have shown that repeated seizures produce neuronal damage and cell death in the hippocampus [44], [45]. Despite the lack of histological studies examining the relationship between recurrent seizures and extrahippocampal remote damage, we know from hippocampal studies that axonal demyelination, formation of axonal spines, increase in interstitial fluid volume due to edema, replacement of axons with glial cells, and astrocyte proliferation may all be associated with the damage caused by seizure activity [46].

Although rodent epilepsy models may differ from human epilepsy, they allow us to study specific pathophysiological mechanisms that are associated with the development and progression of epilepsy in a detailed and controlled manner. The tetanus toxin model is relative mild as compared, for example, to the lithium-pilocarpine [47] and kainate [48] TLE models, that require a prolonged status epilepticus inducing diffuse damage. The functional and structural changes that we found in the tetanus toxin model are therefore more likely to result from frequent seizure propagation alone, rather than a direct effect of tetanus toxin-induced brain damage. This idea is strengthened by the temporal relationship we found between seizure frequency and the changes in graph properties, functional connectivity and fractional anisotropy.

A limitation of our animal study is the necessity to use anesthesia. Isoflurane anesthesia is known to suppress overall functional connectivity in a dose-dependent manner [49]. Although we have demonstrated that low-frequency BOLD fluctuations are largely preserved under light to mild isoflurane anesthesia [50], the correlation of spontaneous BOLD fluctuations during resting-state fMRI acquisition and therefore the strengths of the graph edges and ROI-based functional connectivity may have been lower than under awake conditions.

The TBSS method we used has some disadvantages [51], [52]. TBSS allows, similarly to voxel-based morphometry [53], the comparison of whole-brain maps on a group level, but it is more suited for FA analysis as no spatial smoothing is required. Nonetheless, partial volume effects may still exist [54]. TBSS may also result in wrong estimates in regions with multiple, crossing fiber populations [54]. Another potential drawback of TBSS is the thinning preprocessing step. The thinning procedure causes the statistical analysis to focus on voxels with highest FA only. White matter changes in the lower FA regions of white matter bundles are therefore ignored. These drawbacks may apply to our data as well, although we believe they have a minor impact. The white matter bundles we analyzed do not contain areas with significant crossing fibers. In addition, the major white matter bundles we analyzed in rats are thin structures by nature (i.e., external capsule, corpus callosum, internal capsule). The effect of thinning will therefore be modest.

Unfortunately we were not able to measure EEG simultaneous with MRI acquisition, which is technically challenging and can affect the fMRI quality because of potential artifacts that electrodes would cause on the T_2_^*^-weighted images. This prevents us to rule out any effect of spontaneous seizures on the resting-state fMRI BOLD fluctuations. We do however believe that this effect is unlikely to have happened. We anesthetized the rats during the resting-state fMRI acquisition using isoflurane, which is a potent inhibitor of spontaneous seizures [55]. Directly after each MRI session in all animals, we acquired EEG at identical isoflurane levels and did not observe spontaneous clinical or electrographical seizures. Interictal epileptic spikes were rare. Seizures started to occur only when isoflurane anesthesia was stopped. Therefore we are convinced that functional connectivity, as measured with resting-state fMRI under the anesthetic protocol used in this study, reflects interictal functional connectivity and is not related to frequent spontaneous seizures.

Lastly, although the neocortical tetanus toxin rat model is not related to neuronal cell death [56], direct correlations between gray and white matter MRI measures and histologically measured microstructural integrity are needed. In particular a direct correlation between functional and structural MRI parameters and adaptations at the cellular level will be useful in the characterization of the plasticity process that likely plays a role in brain tissue prone to recurrent spontaneous seizures.

### Conclusion

Taken together, frequent focal seizures induce global abnormalities of white matter and of functional brain networks, characterized by increased functional network segregation and ipsilateral functional connectivity, decreased interhemispheric functional connectivity, and concomitantly increased shortest path lengths, for which spontaneous recurrent seizures may be held responsible. We speculate that increased global network segregation and decreased integration may contribute to cognitive dysfunction in patients with focal epilepsy.

## Materials and Methods

The animal experimental protocol was approved by the Utrecht University Ethical Committee on Animal Experiments. The experiments were carried out in accordance with the guidelines of the European Communities Council Directive. A total of 26, nine weeks old, juvenile male Sprague-Dawley rats (Charles River Laboratories International, Inc., MA, USA), weighing 283±25 g (mean ± SD) at day 0, were included in the study. Animals were group-housed under standard conditions (food and water provided *ad libitum*, 12 h light/12 h dark cycle, temperature 22–24°C).

### Epilepsy model

Chronic focal epilepsy was induced in 13 rats by injection of tetanus toxin (Sigma-Aldrich, Zwijndrecht, The Netherlands) into the right primary motor cortex, which is known to induce frequent, mild facial seizures [25], [26], [27], [28] (epilepsy group). Rats were anesthetized with a subcutaneous (s.c.) injection of a mixture of 0.315 mg/mL fentanyl citrate and 10 mg/mL fluanisone (0.55 mL/kg, Hypnorm®, VetaPharm, Leeds, United Kingdom) and 50 mg/mL midazolam (0.55 mL/kg, Dormicum®, Roche Nederland B.V., Woerden, The Netherlands). During surgery rats were kept warm on a heating pad to prevent hypothermia. A small medial incision was made in the skin covering the skull and the pericranium. A hole in the skull above the right primary motor cortex was made with a 300 μm micro drill. The dura was carefully opened with a micro needle. A volume of 0.6 µL tetanus toxin solution (100 ng/µL) was stereotaxically injected (0.5 nL/min) in the cortex with a 0.5 µL Hamilton syringe with 32 G needle, at coordinates 0.5 mm anterior, 2.5 mm lateral from Bregma and at 1.8 mm depth from the cortical surface. To prevent loss of toxicity, tetanus toxin was dissolved in sterile saline with 0.2% bovine gelatin (Sigma-Aldrich, Zwijndrecht, The Netherlands). After injection the needle was left *in situ* for 15 minutes and removed very slowly and stepwise from the brain. Immediately after surgery, animals were given 0.3 mg/mL s.c. buprenorphine analgesia (0.1 mL/kg, Temgesic®, Schering-Plough Nederland B.V., Houten, The Netherlands). During the next days, if tetanus toxin injected rats became aggressive, they were housed individually. Thirteen age-matched healthy rats served as controls.

Animals were monitored, to detect behavioral changes and clinical seizures, for 30 min at a weekly basis and prior to scan sessions. Seizure activity was defined as behavioral arrest with motor signs, including (a) bilateral whiskers twitching (b) bilateral facial twitching, and (c) facial twitching together with bilateral myoclonic jerks of muscles around the skull and in the neck region [28].

In a random subset of animals (three controls, seven epileptic rats) EEG activity was recorded continually for ten minutes at 1.0% isoflurane outside the MR scanner, immediately following rs-fMRI acquisitions. Bilateral subcutaneous needle EEG electrodes were inserted at the position of the primary motor cortices with a reference electrode above the cerebellum. EEG measurements were conducted using a homebuilt multichannel amplifier, band pass filtered between 0.1 Hz and 250 Hz, a National Instruments™ NI USB-6211 DAQ with a sampling rate of 1000 Hz per channel and LabWindows™ programmed data acquisition software. After ten minutes, isoflurane anesthesia (which is known to suppress epileptic activity [57]) was lowered to 0%, while EEG monitoring and mechanical ventilation continued, until animals woke up. EEGs were visually inspected for interictal spiking, and for the occurrence of electrographical seizure discharges, being previously defined as series of rhythmic EEG spikes [28].

### Structural and functional MRI

All MRI experiments were performed on a 4.7 T SISCO/Varian system (Palo Alto, CA, USA) at seven, 21, 49 and seventy days after epilepsy induction. Radiofrequency excitation and signal detection were accomplished with a Helmholtz volume coil (diameter, 9 cm) and an inductively coupled surface coil (diameter, 2.5 cm), respectively.

Before MRI the animals were endotracheally intubated and mechanically ventilated with 2.0% isoflurane in a mixture of O_2_/air (1/2 volume/volume; 55 beats/min). During MRI, expired CO_2_, blood oxygen saturation and heart rate were continuously monitored and kept within physiological levels. A feedback-controlled heating pad was used to maintain body temperature at 37.0±0.5°C.

First, multiecho multislice T_2_-weighted MRI [repetition time (TR)/echo time (TE), 3000/17.5 ms; 19 coronal slices; field of view (FOV), 32×32 mm^2^; acquisition matrix, 128×128; voxel resolution, 0.25×0.25×1.0 mm^3^; echo train length, 12] was conducted to assess possible changes in brain T_2_ relaxation times.

Second, a gradient echo T_2_^*^-weighted 3D dataset was collected for registration purposes [TR/TE, 10/2.57 ms; FOV, 40×40×40 mm^3^; data matrix, 128×128×256; voxel resolution, 0.313×0.313×0.234 mm^3^; pulse angle, 20°].

Third, high angular resolution diffusion imaging [TR/TE, 3500/26 ms; 25 transverse slices; FOV, 32×32 mm^2^; acquisition matrix, 64×64; voxel resolution, 0.5×0.5×0.5 mm^3^; δ, 6 ms; Δ, 11 ms; b-value, 1184.33 s/mm^2^; non-diffusion weighted images, 2] was acquired to assess microstructural white matter integrity. A spherical acquisition scheme with fifty unique gradient directions, determined with electrostatic repulsion [58], was used.

Finally, repetitive BOLD MRI was conducted [TR/TE, 500/19 ms; 7 coronal slices; FOV, 32×32 mm^2^; acquisition matrix, 64×64; voxel resolution, 0.5×0.5×1.5 mm^3^; pulse angle, 35°; temporal resolution, 500 ms; number of scans, 1200; total scan time, 10 min] using a gradient echo single shot EPI sequence. Exactly ten minutes prior to rs-fMRI acquisition, end-tidal isoflurane anesthesia concentration was reduced to, and maintained at 1.0%. At this level of isoflurane anesthesia, coherence of low-frequency BOLD signal fluctuations between functionally connected regions has been shown to be preserved [50].

### Preprocessing

After bias-field inhomogeneity correction [59] and masking out nonbrain structures [60], the T_2_^*^-weighted 3D volume was nonlinearly registered to the a stereotaxic rat brain atlas [61]. Next, BOLD MR images were linearly registered with the T_2_^*^-weighted 3D image. Registrations were performed with the elastix toolkit (http://elastix.isi.uu.nl; [62]). Matching of MR images with the atlas and the functional images allowed functional anatomy-based delineation of (bilateral) ROIs. We combined the primary and secondary motor cortices, and the fore- and hindlimb region of the primary somatosensory cortices to create a single sensorimotor cortical ROI in the left and right hemispheres [63], [64].

### Resting-state fMRI analysis

Resting-state fMRI enables the assessment of functional connectivity in the brain. If neuronal signaling between two areas – measured as low-frequency BOLD fluctuations – is temporally coherent, these areas are considered to be functionally connected. Several methods exist to calculate such temporal coherency. We used the Pearson's correlation coefficient *r*. We subsequently normalized *r* into *z*' using the Fisher's *z*'-transformation [65]. Non-neuronal signal contributions were minimized by means of spatial smoothing (the smoothing kernel full-width at-half-maximum was set to 1.0 mm), band-pass filtering (between 0.01 and 0.1 Hz) and linear regression with nuance signals, including the mean brain BOLD signal oscillations, the mean signal form the white matter, the mean signal from the cerebrospinal fluid, and the rotation and translation parameters as obtained from the motion correction [64]. After these preprocessing steps, two rs-fMRI analyses were performed; (a) a global network analysis, including all cortical and subcortical voxels, and (b) a ROI-based analysis in left and right sensorimotor cortices.

#### Network analysis

Each functional dataset was considered as a weighted undirected network, described by the graph *G*  =  (*V*, *W*), where *V* is the number of nodes and *W* is the collection of edges *w_ij_* is the *V*×*V* symmetric weight matrix, where *w_ii_* = 0. In our data, *V* was the collection of *N* cortical and subcortical gray matter voxels and *w_ij_* the normalized correlation coefficient *z*', defined between voxel time series *i* and *j*. *N* varied slightly between animals and time points (mean ± SD: 2003±126). Edges with negative correlation values were set to 0.

We quantified the local and global graph structures via the weighted undirected clustering coefficient [66] and the shortest path length [67], using the C++ Boost Graph Library (www.boost.org; [68]).

The overall clustering coefficient was defined as:
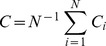



with the clustering coefficient for node *i*:
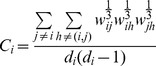



Taking into account weights of all edges in a triangle, but not considering weights not participating in any triangle. *L* is defined as the mean geodesic length over all couples of nodes:
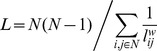



with 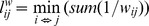.

The harmonic mean approach avoided inclusion of disconnected nodes in calculating *L* and resembles the global efficiency measure (i.e. 1/∞ → 0) [69]. For each functional dataset, *L* and *C* were normalized using 100 surrogate networks. This number was sufficient to result in stable surrogate network properties (data not shown). Surrogate networks were constructed using the random rewiring procedure described in [70]. Normalized weighted *L* and *C* were defined as:
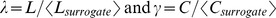



#### Region-of-interest analysis

To identify the basis of changes in global functional network topology we also performed three different ROI-based analyses: (a) Correlation of the average sensorimotor cortical signal, for left and right ROIs separately, with all brain voxels. The obtained connectivity maps were conservatively thresholded at *z*'>0.15 and overlaid on an anatomical template; (b) correlation of the average left and right sensorimotor cortical ROI signals, as a measure of interhemispheric functional connectivity [64]; (c) for each hemisphere separately, calculation of total volume of all voxels that correlated significantly (i.e., *z*'>0.15) with the average signal from the sensorimotor ROI within that hemisphere, as a measure of intrahemispheric functional connectivity.

### Structural MRI

#### Diffusion tensor imaging analysis

The acquired high angular resolution diffusion scans were registered to the average non-diffusion weighted image with an affine transformation to correct for head motion and eddy-current distortions, and brain tissue was masked out [60]. The set of gradient vectors was adjusted according to the rotation of the individual scans. The average non-diffusion weighted image was matched with the T_2_^*^-weighted 3D dataset using affine registration. Next, the effective diffusion tensor, the corresponding eigensystem, and the subsequently derived FA, ADC_trace_, and axial and radial diffusivity maps were computed for each voxel [71]. A total of forty control FA maps were nonlinearly registered to a common reference to construct a FA template. Next, localized statistical testing of FA data was carried out using TBSS [51], [52]. TBSS overcomes many of the problems inherent to conventional voxel-based morphometry analysis [53], potentially resulting in spurious findings if spatial misalignment is present [52]. Using TBSS, all registered FA maps were averaged, ‘thinned’, and individual FA values were projected onto this thinned white matter skeleton and fed into voxel-wise randomization testing. In addition to the TBSS analysis, a ROI analysis was carried out. The corpus callosum was manually outlined on the FA template, to calculate its FA, ADC_trace_, and axial and radial diffusivity values.

### Statistical evaluation

Repeated measures linear mixed models [72] were employed to characterize changes in graph properties, inter– and intrahemispheric functional connectivity and tissue diffusion measures over time.

Random group and time effects, first order interaction between time and group, and continuous AR1 correlation structure [73] were added to the model. The model parameters were estimated by the restricted maximum likelihood method and considered significant if *p*<0.05 (corrected using Tukey's method). All statistical analyses were performed in R (www.r-project.org; [74]) using the nlme package [75]. Voxel-wise statistical analysis of the FA data was carried out using permutation *t*-testing and thresholded at *p*<0.05 (false discovery rate corrected [76]).
